# Intracranial progression of the epidermoid cyst to a white epidermoid cyst in a pediatric patient: a case report

**DOI:** 10.1186/s13256-023-04243-y

**Published:** 2023-11-29

**Authors:** María Fernanda Laverde-Reyes, Juan Camilo Márquez, Daniela Nasner, Ana María Granados-Sánchez, Luis Fernando Castillo

**Affiliations:** 1 Department of Radiology and Diagnostic Imaging, Faculty of Health Sciences, Universidad Icesi, Cali, Colombia; 2 Neuroradiology Section, Department of Radiology and Diagnostic Imaging, Fundación Valle del Lili, Cali, Colombia; 3 Clinical Research Center, Fundación Valle del Lili, 760032 Cali, Colombia

**Keywords:** Epidermoid cyst, White epidermoid cyst, Diffusion-weighted imaging, Brainstem

## Abstract

**Background:**

Epidermoid cysts are rare benign lesions that originate from remnants of ectodermal epithelial tissue, particularly infrequent in the pediatric population. They exhibit characteristic imaging features, with occasional variations leading to the development of a “white” epidermoid cyst. This transformation results from the presence of protein and lipid material within the cyst, causing intrinsic hyperintensity in T1-weighted images, signal hypointensity in T2-weighted images, and a bright signal in diffusion-weighted imaging.

**Case presentation:**

We describe the case of a 5-year-old Latina pediatric patient initially diagnosed with a typical epidermoid cyst. After 13 years of follow-up, this typical epidermoid cyst underwent a transformation, becoming a “white” epidermoid cyst.

**Conclusions:**

Epidermoid cysts are rare intracranial lesions. The term “white epidermoid cyst” does not denote a variant; it represents a distinct transformation within an epidermoid cyst due to liquid and protein accumulation. This transformation should be considered in cases with specific imaging characteristics.

## Background

Epidermoid cysts are lesions that originate from remnants of ectodermal epithelium during the closure of the neural tube between the third and fifth weeks of pregnancy [1]; these are usually congenital lesions, accounting for less than 2% of all primary intracranial tumors, and they tend to be asymptomatic [2, 3]. However, in some cases, they may contain a more significant protein and lipid component [6], leading to changes in signal intensity, such as T1 intrinsic hyperintensity and low signal intensity in T2 sequences, which are known as white epidermoid cysts [5]. White epidermoid cysts make up approximately 3% of all epidermoid cysts [7].

In this report, we present a rare clinical case of a pediatric patient who was initially diagnosed with a typical epidermoid cyst in the prepontine region. Subsequent imaging follow-up revealed the evolution of the cyst into a white epidermoid cyst.

## Case presentation

**Fig. 1:**
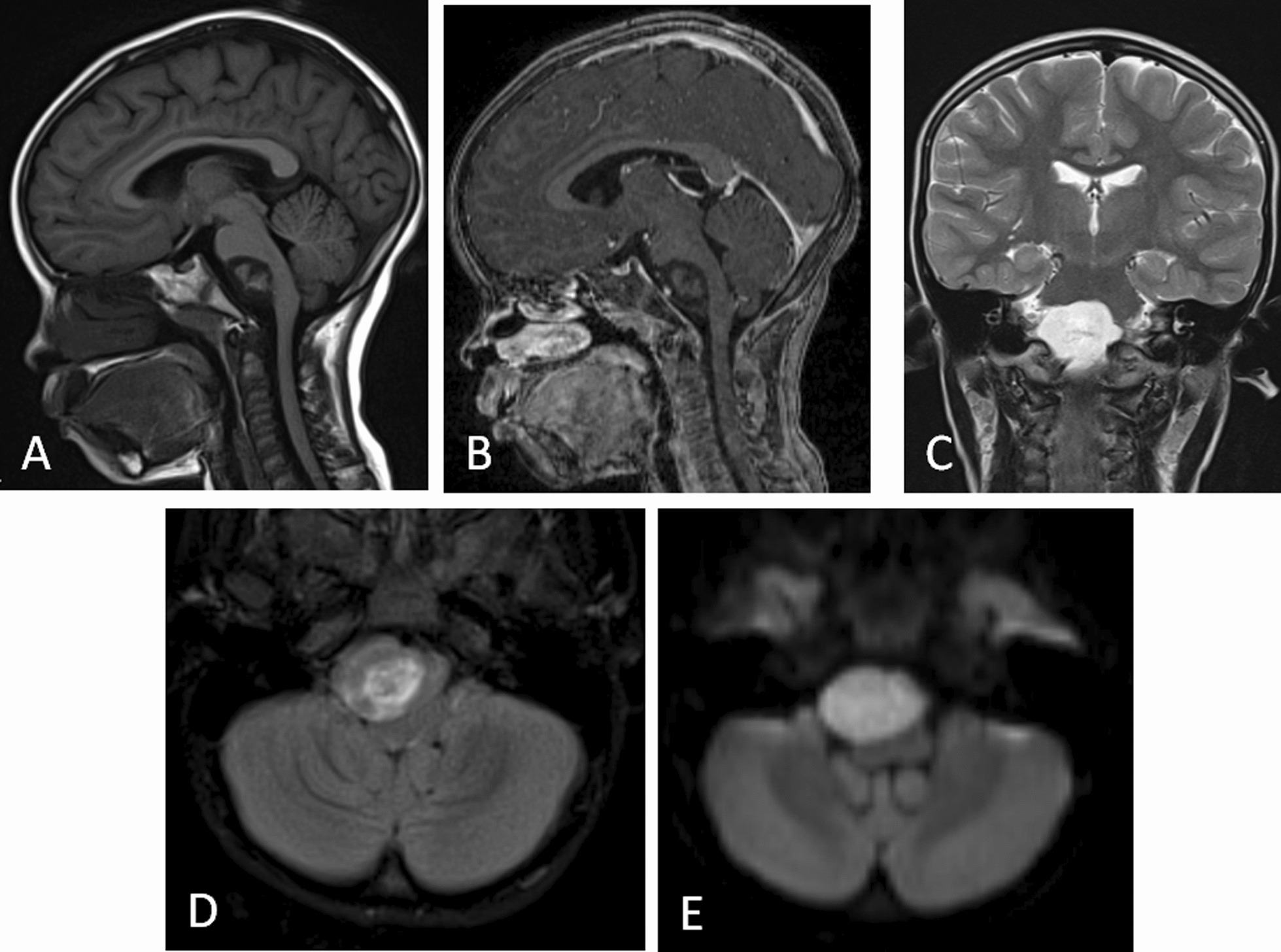
MRI with contrast. **A** Sagittal T1 reconstruction; prepontine extraaxial mass with hypointense behavior. **B** Sagittal T1 reconstruction with contrast; no enhancement of the mass observed. **C** Coronal T2; mass with intensity similar to cerebrospinal fluid (CSF). **D** Axial FLAIR scan showing lobulated heterogeneous predominantly hyperintense mass giving the appearance of “dirty FLAIR”. **E** Axial view on DWI showing bright mass

The patient is a female with a history of headaches and a single seizure episode at the age 5 years old. She underwent contrast-enhanced head magnetic resonance imaging (MRI), which revealed the presence of a prepontine extraaxial mass with predominantly hypointense signal intensity in T1, similar to cerebrospinal fluid (CSF) in T2. Additionally, there was heterogeneity in fluid attenuated inversion recovery (FLAIR) imaging, giving the appearance of “dirty FLAIR.” The mass appeared bright in diffusion weighted imaging (DWI) and did not show enhancement after the administration of contrast medium, findings consistent with an epidermoid cyst (Fig. 1).

According to the patient’s mother, she was followed up with neurology at another facility because she continued to experience weekly headaches with a frontoparietal location, rating the pain at 8/10 on the pain scale. Although these headaches did not significantly affect her quality of life and lasted only for a few minutes, they occurred without any focal neurological deficits.

**Fig. 2:**
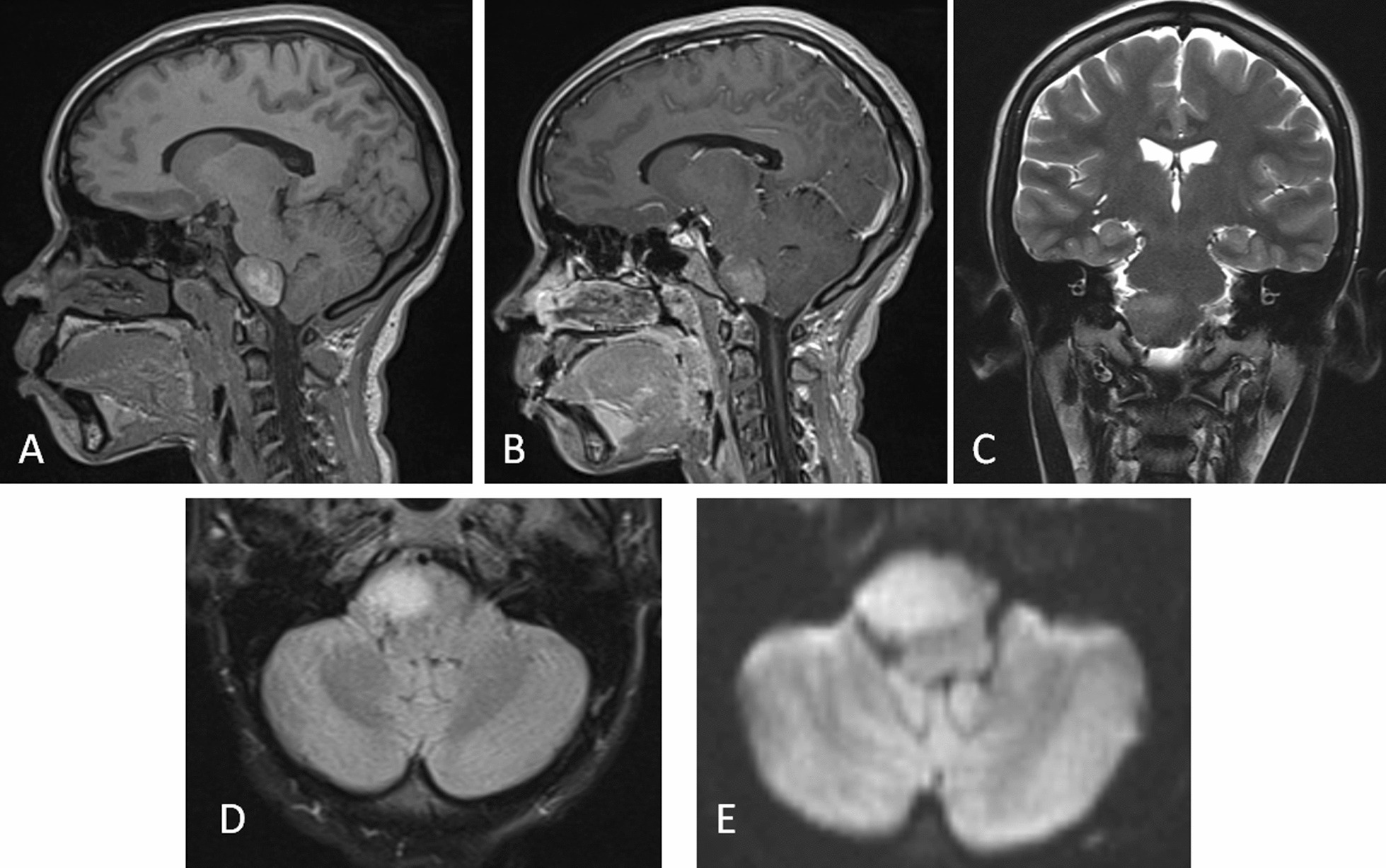
Brain MRI with contrast. **A** Sagittal T1 reconstruction with persistence of prepontine extraaxial mass that, when compared with previous MRI, had increased in size proportionally to the rest of the head and presented with heterogeneous signal intensity due to protein and lipid component that was not previously visualized. **B** Sagittal T1 contrast-enhanced sagittal reconstruction. The mass does not show enhancement after medium contrast administration. **C** Coronal T2 heterogeneous mass predominantly hypointense; it no longer preserves CSF-like signal intensity. **D** Axial FLAIR section with heterogeneity in signal intensity, although predominantly hyperintense. **E** Axial slice on DWI showing persistence of bright mass

The patient sought consultation at our institution at the age of 18 years due to persistent headaches. Neuroimaging was performed, revealing no significant changes in the size or location of the mass. However, the magnetic characteristics of the lesion had undergone a transformation, now corresponding to the evolution of the cyst into a “white epidermoid cyst” (Fig. 2).

The patient is currently under conservative outpatient neurosurgical care and continues to experience only occasional headaches without any neurological deficits. It is worth noting that she had a single seizure episode at the age of 5 years.

## Discussion

Epidermoid cysts are lesions originating from a remnant of ectodermal epithelium during the closure of the neural tube between the third and fifth week of pregnancy [1]. These cysts are typically congenital and represent less than 2% of all primary intracranial tumors [2, 3]. While they are usually asymptomatic, larger cysts can exert pressure on adjacent structures, leading to symptoms like headaches and seizures [2]. The prevalence is highest in the third and fourth decades of life [4], with many patients, including adults, remaining asymptomatic. Regarding location, epidermoid cysts most frequently appear in the subarachnoid space at the cranial base, often involving the pontocerebellar angle and areas adjacent to the sellar region [5].

Epidermoid cysts are well-defined lesions typically exhibiting hypointensity in T1-weighted sequences, hyperintensity in T2-weighted images with signal intensity similar to cerebrospinal fluid (CSF), and hyperintensity in DWI [2]. Infrequently, these cysts can also contain more significant protein components, keratin, lipids, and cholesterol [6], leading to changes in signal intensity, resulting in hyperintensity in sequences with T1 information and low signal intensity in T2 sequences. This variant is known as a “white epidermoid cyst” [5]. Reviewing the literature reveals that this entity accounts for approximately 3% of all epidermoid cysts [7], and some studies have reported a slight predilection for females [2].

It is important to note that in brain computed tomography (CT), an epidermoid cyst typically appears hypodense. However, it is crucial to understand that the presence of hyperdensity in CT does not necessarily indicate a “white” epidermoid cyst, as there is not always a correlation between hyperdensity in CT and hyperintensity in MRI in T1 sequences. Braun *et al.* described this as an atypical representation of dense epidermoid cysts in 1977, attributing their etiology to areas of saponification and calcifications [8].

Conservative treatment has demonstrated promising results with minimal complication rates, while surgical excision is the preferred treatment for symptomatic cases. However, complete resection of epidermoid cysts poses a significant challenge for neurosurgeons and may carry a high risk of morbidity and mortality [4]. It can be particularly difficult to remove all cyst tissue, especially in areas surrounding cranial nerves and blood vessels. As a result, recurrence is not uncommon, although the growth is typically slow, and many years can elapse without new symptoms.

## Conclusions

Epidermoid cysts are infrequent benign intracranial lesions. It is important to clarify that the term “white epidermoid cyst” does not refer to a variant of an epidermoid cyst; rather, it signifies a specific transformation that can occur within an epidermoid cyst due to liquid and protein degeneration. Therefore, it is essential to consider the possibility of this transformation in cases where well-delimited extraaxial lesions exhibit hyperintense behavior in T1 with decreased signal intensity in T2, along with a bright intensity in DWI.

## Data Availability

Not applicable.
